# Explaining the Consumption Technology Acceptance in the Elderly Post-Pandemic: Effort Expectancy Does Not Matter

**DOI:** 10.3390/bs13020087

**Published:** 2023-01-21

**Authors:** Patricio Ramírez-Correa, Elizabeth Eliana Grandón, Muriel Ramírez-Santana, Jorge Arenas-Gaitán, F. Javier Rondán-Cataluña

**Affiliations:** 1 School of Engineering, Universidad Católica del Norte, Coquimbo 1781421, Chile; 2 Department of Information Systems, Universidad del Bío-Bío, Concepción 4051381, Chile; 3 Public Health Department, Faculty of Medicine, Universidad Católica del Norte, Coquimbo 1781421, Chile; 4 Department of Business Administration and Marketing, Universidad de Sevilla, 41004 Sevilla, Spain

**Keywords:** consumer technology, elderly, effort expectancy

## Abstract

Consumer technology has been enormously boosted by the COVID-19 pandemic, with one of the primary consumers being the elderly. In this scenario, it is necessary to consider the impact of technologies on different older generational cohorts to understand the future of a data-driven digital society fully. This research aims to explain the acceptance of social networking sites, a particular consumer technology, in the post-pandemic elderly population. Data were obtained from 1555 older adults in Chile based on a consumer technology acceptance model. The respondents were grouped according to their technological predisposition and their generation into three groups. Applying a multigroup analysis based on structural equation modelling reveals significant differences in the explanatory variables of the intention and use of this technology between the groups. And more remarkably, the effort expectancy is not statistically significant as a variable to explain this acceptance globally in either of the three groups. There are two principal contributions of this study. First, it shows why adults adopt consumer technology after the pandemic. Second, it validates a classification of elderly adults who use consumption technologies that are useful in understanding the heterogeneity of this phenomenon.

## 1. Introduction

Internet and online applications are becoming increasingly popular, with Internet users accounting for 65.6% of the world’s population. To a certain degree, the Internet and various online applications are an essential part of our daily lives. In this context, behavioural scientists are asked to look at the regular use of digital technology in everyday life [1]. In response to this request and considering the significant generational gap that hinders the usage of digital technology [2], understanding the predictors of this technology usage behaviour in older adults is relevant. Earlier studies have explained the use of digital technology in older adults based on sociodemographic, attitudinal, functional, and physical factors. However, in these studies, older adults were considered one block [3]. The current study closes this gap by focusing on the differences between older user segments of digital technologies rather than viewing them as a homogeneous block.

In our digitised society, the use of digital media is on the rise. It has been significantly boosted by the COVID-19 pandemic, mostly in previously excluded segments such as older generations [4], enabling innovative service solutions for industry 5.0 aimed at these groups of consumers [5]. Social networking sites (SNS), a particular consumer technology, have been at the centre of this growth. Research should consider the impact of technologies on different older generational cohorts to fully understand the future of a data-driven digital society [3]. In this vein, before the COVID-19 pandemic, Nunan and Di Domenico [6] identified that the generational cohorts’ aspects in using SNS require more research. Recently, Berg and Liljeda [7] highlighted the need for studies considering generations when analysing older adults as consumers. 

In the last decade, various studies have shown that older people benefit from using SNS by improving their wellbeing. Among the reasons, it is mentioned that SNS facilitate social inclusion [8] contribute to increasing social capital [9], improve cognitive processes [10], self-esteem and the feeling of belonging to a group [11], and eventually, take advantage of new ways of marketing [12]. The manifestations of these benefits have increased after the experience of confinement lived worldwide during the restrictions imposed to control the COVID-19 pandemic. In the last two years, the literature on the subject has grown, showing an expansion in the use of SNS by older people and its impact on reducing isolation and loneliness during lockdown periods [13,14,15,16,17]. Some authors even ventured to suggest interventions of various kinds to reduce the isolation caused by social restrictions and thus contribute to the wellbeing of the elderly [17]. Among these interventions is promoting the use of SNS in this age group [18], which have been recognised as tools that improve wellbeing in the context of a pandemic [14,15,16,19,20].

In this context, our study aims to explain the SNS acceptance in the elderly post-pandemic. We use two theoretical frameworks to achieve this purpose: the unified theory of acceptance and use of technology version 2 (UTAUT2) model and the technology readiness index (TRI). Based on these frameworks, we study a structural equation model and analyse differences between elderly groups associated with different generations and technological predispositions. The main contribution study is twofold. First, it shows how adults embrace consumer technologies after the pandemic. Second, it validates a classification of older adults who use helpful consumer technologies to understand the heterogeneity of this phenomenon.

The rest of this paper comprises the literature review, the development of the hypotheses, the description of materials and methods and the results. The article ends with a discussion of the findings and conclusions.

## 2. Literature Review

### 2.1. UTAU2 and Elders

The unified theory of acceptance and use of technology (UTAUT) [21] has been used to explain social behaviour in information systems research. This theory combines concepts from different approaches to explain behavioural intention, use, and acceptance of new technology. Behavioural intention is the degree to which a person has formulated plans to perform some specific behaviour [22]. The theory sustains that the intention to achieve a particular behaviour, particularly information technology (IT) adoption, depends on four primary factors: performance expectancy, effort expectancy, social influence, and facilitating conditions. The theory also hypothesises that the intention to perform a behaviour and facilitating conditions that may be in place to exert the behaviour influences actual behaviour. 

Venkatesh and colleagues define performance expectancy as the degree to which the use of technology will benefit people in the execution of some tasks [21]. Studies have found a significant direct relationship between performance expectancy and technology adoption among the elderly. For instance, Ref. [23] found that performance expectancy was the most important factor that positively influenced the intention to use a modern automated driver assistance system and full driving automation. Similarly, Ref. [24] found that performance expectancy significantly influences the gameplay intention of the elderly. 

Venkatesh and colleagues define effort expectancy as the degree of ease associated with the use of technology by people [21]. According to [25], effort expectancy captures older adults’ perception of the extent to which they feel comfortable using social media. Effort expectancy has been found to significantly affect the intention to use technology in the elderly. For instance, Ref. [26] found that effort expectancy influences older adults’ acceptance and use of information and communication technology. Other studies have found that this construct does not affect the intention to use technology [27,28,29].

Based on Ajzen and Fishbein’s study [22], Venkatesh and colleagues describe social influence as the degree to which an individual perceives that referent people believe they should use a particular technology [21]. Some studies have found a significant effect of social influence in predicting different types of technology adoption among the elderly. For example, Ref. [30] found that the major factors driving older adults toward online shopping are social influence and performance expectancy. 

Finally, Venkatesh and associates define facilitating conditions as individuals’ perception of having the resources and support to perform a behaviour technology [21]. Facilitating conditions is a key predictor in the UTAUT model and has been widely studied in the adoption of technologies by the elderly, including the Internet, healthcare technology, and online shopping [31]. Nevertheless, the effect of this factor could be more consistent. For instance, Ref. [26] observed a significant impact in facilitating conditions for older people to anticipate Internet adoption; on the contrary, Ref. [32] found an insignificant effect of facilitating conditions in predicting wearable health technology adoption for older people.

The constructs of the UTAUT model have been vital in explaining the intention to use diverse technologies among elders. For instance, Hoque and Sorwar found support to indicate that performance expectancy, effort expectancy, and social influence impact the intention to use m-Health by the elderly [33]. In an analogous and recent study, Tian and Wu revealed that effort expectancy, performance expectancy, social influence, and facilitating conditions could directly and significantly influence the continuance intention of mobile health of elders with chronic diseases [34]. Based on the studies mentioned above, this research proposes the following hypotheses:

**Hypothesis** **1** **(H1):** *Performance expectancy positively influences older Chilean adults’ intention to use SNS*.

**Hypothesis** **2** **(H2):** *Effort expectancy positively influences older Chilean adults’ intention to use SNS*.

**Hypothesis** **3** **(H3):** *Social influence positively influences older Chilean adults’ intention to use SNS*.

**Hypothesis** **4** **(H4):** *Facilitating conditions positively influence older Chilean adults’ intention to use SNS*.

To advance the explanation of the theory of massive consumption technology, Venkatesh and associates added three new determinants of behavioural intention: hedonic motivation, price-value, and habit; this last construct also being directly related to the use of technology [35].

Venkatesh and associates stated that hedonic motivation is the fun or pleasure an individual experiences when using technology [35]. The literature has highlighted the importance of hedonic motivation in determining an individual’s technology adoption [32,36]. For example, elderly users who enjoy healthcare technology products are more likely to use them [32].

Price–value is a factor that may address the cost concern of using the technology and directly determines an individual’s intention to use technology [31]. Venkatesh and colleagues stated that price–value is the individual’s cognitive compensation between the perceived benefits and the monetary cost of using technology [35]. In exergaming technology, price–value has been shown to affect the elderly’s intention to use technology directly [37].

Habit is a critical predictor with a moderating effect, direct and indirect, of a person’s behavioural intention to use technology [31]. Venkatesh and associates stated that habit is the degree to which individuals tend to perform automatic behaviours due to learning [35]. Regarding Internet adoption, habit has been shown to directly affect the elderly’s intention to use the Internet and both direct and indirect effects on the elderly’s use behaviour [26]. Furthermore, habit has been moderately associated with the behavioural intent of older adults to use an exergaming technology [37].

This new model, UTAUT2, has become a robust theoretical model for predicting IT acceptance. For example, Palas and colleagues found that hedonic motivation, price–value, and habit significantly impacted the elders’ behavioural intention to adopt m-Health services [38]. Similar results were found by Yein and Pal when analysing user acceptance of exergaming (fall-preventive measure) tailored for the Indian elderly [37]. Nevertheless, the literature points out an insignificant effect of price–value when users perceive that the service offered is free [39]. In particular, the monetary cost of having an SNS is included in the cost of having Internet access. Therefore, it is difficult to evaluate the pure effect of price–value since the business model of SNS providers considers the content generated by users as a profit source. For this reason, since in previous research on older populations, price–value has not been found as a significant antecedent of behavioural intention [40], we did not consider it in the research model. Given the above, this study states the following hypotheses:

**Hypothesis** **5** **(H5):** *Hedonic motivation positively influences older Chilean adults’ intention to use SNS*.

**Hypothesis** **6** **(H6):** *Habit positively influences older Chilean adults’ intention to use SNS*.

**Hypothesis** **7** **(H7):** *Habit positively influences older Chilean adults’ use of SNS*.

**Hypothesis** **8** **(H8):** *Facilitating conditions positively influence older Chilean adults’ use of SNS*.

**Hypothesis** **9** **(H9):** *The intention to use SNS is positively related to older Chilean adults’ use of SNS*.

### 2.2. Generations and Technology Readiness as Moderators of Information Technologies Acceptance

#### 2.2.1. Generations as Moderators of Information Technologies Acceptance

The generational cohort theory was coined by Karl Mannheim [41] and developed by other researchers [42,43]. In general terms, a generation refers to a period where identity is built on resources and meanings that are socially and historically available. In this sense, a generation can last for several centuries, as occurred in pre-modern societies, or for only a decade. The generation ends when historical events empty the previous system and its associated social experiences of meaning [43]. 

In the technology field, an individual belonging to a generation has been used as a moderator variable in acceptance models. That is, it has been proposed that having a socially and historically distinct identity changes the effects independent variables have on the dependent variables in the acceptance phenomenon. Regarding technology in general, Calvo-Porral and Pesqueira-Sanchez [44] studied whether the generational cohort influences technology behaviour. Based on the uses and gratifications theory, they analysed data from individuals residing in Spain on a random basis. Specifically, this study compared responses from 707 Millennials and 276 Generation X individuals. Their results suggest a moderating role of the generational cohort in using technologies. Millennials mostly use and become engaged with technologies for entertainment and hedonic purposes, while utilitarian purposes and information search mainly drive Generation X individuals.

More specifically, some authors have explored the moderation of generations in digital technologies. Noah and Sethumadhavan [45] examined trust in digital assistants for three generations (generations X, Y, and Z). Analysis of the sample of 278 regular users of digital assistants, primarily from the U.S., showed that Gen Z trusted digital assistants more than Gen X. In addition, there are different sets of trust predictors per generation. Bordonaba-Juste and colleagues [46] examined intergenerational differences in the intent to pay for cloud services. The analysis showed differences across generation cohorts based on secondary data of 2480 Spanish cloud users. For instance, ubiquity, data loss protection, and ease of sharing explain that the Baby Boomer generation pays for cloud services.

Notwithstanding, among Gen Y, access to more significant online resources and privacy concerns are important aspects of explaining paying for cloud services. In Gen X, previous negative security experiments have a more substantial impact on the probability of payment. Debb and associates [47] compared the information security behaviours of Gen Y and Gen Z based on the measurement of online security behaviours. Based on a sample of 593 individuals from two universities in the USA, their findings indicated significant differences between Gen Y and Gen Z. Gen Y reported a more thorough review of privacy policies on social media, maintenance of antivirus updates, monitoring unusual computer performance, and implementing malware alerts. The authors inferred that this generation was more experienced and less likely to view themselves as invulnerable to online victimisation. Lastly, Alkire and colleagues [48] explored how the adoption of healthcare patient portals is affected by generational cohorts. The study collected 268 patient responses, of which 138 were from Gen X and 130 were from Gen Y. The results indicate differences in strength in the relationships of the proposed technology acceptance model associated with the generational cohorts. Based on their findings, these authors suggest that digital technology should be designed and implemented in the context of generational cohorts. 

Additionally, some studies have reviewed how generations moderate the acceptance of commerce services. Sharma and associates [49] examined differences in Australian consumers’ intention to purchase travel online among Gen Y and Baby Boomers based on a technology acceptance model. Their findings showed that the factors influencing consumers’ intent to buy travel online differed across generations and underscored the importance of generational cohort theory in intending to purchase travel online. Lissitsa and Kol [50] explored the association of personality traits and mobile shopping intentions for hedonic products across generational cohorts. Based on data from 1241 Israeli Jews belonging to four generations (Gen Z, Gen Y, Gen X, and Baby Boomers), different personality traits were associated with mobile shopping intention among the different generations. For example, a positive association between openness to experience and mobile shopping intention was uncovered among Baby Boomers and Gen X; extraversion was positively correlated with mobile shopping intention among Gen Y; a negative correlation between agreeableness and mobile shopping intention was observed among Gen Z. Moreover, Çera and colleagues [51] explored the role of generational cohorts in mobile banking adoption based on a technology acceptance model. Using data from 959 individuals from southeastern Europe, the findings indicated that the effects to explain the intention to use mobile banking were bigger for Gen Y than Gen Z. There were no differences between Gen X and Y. Finally, Agárdi and Alt [52] compared Gen X and Gen Z regarding mobile payment acceptance. A sample of 580 Hungarian citizens owning smartphones was analysed, revealing that the perceived ease of use, subjective norms, and financial risk of mobile payment influenced Gen Xs. In turn, Gen Zs intended to make greater use of mobile payments if they perceived it to be compatible with their way of life. 

Research must consider the impact of technologies on different older generational cohorts to understand the future of a data-driven digital society fully. In this vein, Nunan and Di Domenico [6] executed an extensive literature review of research gaps concerning how older people adopt new digital technologies. Among these gaps, they identified that the generational cohorts’ aspects in using social networking sites require more research. In the age segment of older adults, three cohorts have recently been proposed: silent, early Baby Boomers, and late Baby Boomers [53,54,55,56]. In specific, older Chilean adults include these three cohorts: the silent generation, people born between 1927 and 1946; early Baby Boomers, individuals born between 1947 and 1955; and late Baby Boomers, people born between 1956 and 1961. A distinction between early and late baby boomers can be made based on the areas that most affect Chilean generations: technology, family, economy, sexuality, and politics [57]. Concerning technology, although the two cohorts are digital immigrants, there are noticeable differences. Access to personal computers in the 1980s found the early Baby Boomers as young adults while the late Baby Boomers were still young.

On the other hand, the advent of the Internet to the public in 1999 found the two cohorts as adults. Still, the massification of the Internet, with rates of use increasing more than 50% in 2011, saw the early Baby Boomers as older people; in turn, late Baby Boomers were still adults. Concerning family, the two cohorts follow traditional guidelines; for example, although the cohabitation rate has tripled in the last three decades, at the age of formalising their affective relationships, the cohabitation rates for these two cohorts were below 6%. Furthermore, the birth rates are similar in the fertile age of the two cohorts. The 1985 economic crisis affected both cohorts as young adults, hurting their job opportunities. However, the early Baby Boomers already had memories of the 1975 financial crisis as workers; the late ones did not. Then came the economic boom of the 1990s that prompted the late baby boomers as young adults to take risks and grow economically; the early ones were already closer to adulthood and possibly took less risk. On the other hand, access to higher education was at meagre rates. In general, only their children enjoyed increased university coverage from 7.5 in 1980 to 15.6 in 1990 and 53.1 in 2015. However, a group from the late Baby Boomers, thanks to the increase in the supply of higher education, were able to study as young adults in workers’ programs, which opened to them new economic opportunities.

Regarding sexuality, we can highlight that the hippie movement occurred when the early baby boomers were young. Therefore, they could be more permeable to free love tendencies; instead, the late baby boomers were still children. On the other hand, the first case of AIDS in 1984 impacted the late baby boomers as young people, and in the case of the early, they were already young adults. Undoubtedly, this fact must have affected the sexual behaviour of the former, mainly without a stable sexual partner. Regarding politics, in the global context, the end of the Cold War was found early and late as adults and young adults, respectively; the two cohorts have memories of this polarization. There is an essential differentiating element in the Chilean context. During the 17 years of the military dictatorship (1973–1990), the early Baby Boomers were young adults with their own family and work responsibilities. On the other hand, the late Baby Boomers were young and suffered severe limitations on freedom of movement and expression. In summary, in Chile, the early Baby Boomers are close to the silent generation, and the late Baby Boomers differ from those two cohorts.

#### 2.2.2. Technology Readiness Index as Moderator of Information Technologies Acceptance

Parasuraman [58] developed the technology readiness index (TRI), a scale for measuring people’s propensity to adopt and use new technological products and services. The TRI is much more a reflection of people’s mental attraction to—or avoidance of—technology-based systems than of people’s technical ability. Parasuraman’s TRI contains four factors: optimism, innovation, insecurity, and discomfort. Optimism is the positive vision of technology and the belief that it provides people with more control, flexibility, and effectiveness. Innovativeness is the trend toward being a technology pioneer and opinion leader. Discomfort is the perceived lack of control over technology and feeling overwhelmed by it. Lastly, insecurity is mistrust of technology and scepticism about its ability to operate correctly. TRI can also segment consumers based on technology readiness into five profiles: explorers, pioneers, sceptics, hesitators, and avoiders.

Several studies have integrated technology acceptance models and TRI. Lin and colleagues [59] combined the TRI into the technology acceptance model (TAM) to explain e-services adoption. Based on data from 406 consumers, the results indicated that TRI was a causal antecedent of both perceived usefulness and perceived ease of use but did not directly affect the consumers’ intentions to use e-services. Other authors followed the same idea, Hallikainen and Laukkanen in accepting digital B2B services [20], and Larasati and associates in using ERP systems [60]. Kim and Chiu [61] applied a similar idea to explain the consumer acceptance of wearable sports technology. Still, they divided TRI into positive (optimism and innovativeness) and negative (discomfort and insecurity) factors. Their findings, based on data from 300 users of wearable sports technology, indicated that the positive factor of the TRI was a positive antecedent of both perceived usefulness and perceived ease, and the negative factor of the TRI was a negative antecedent of both perceived usefulness and perceived ease. Additionally, TRI has been found as the antecedent of the constructs of the unified theory of acceptance and use of technology (UTAUT). For instance, Seol and colleagues [62] and López-Pérez and associates [63] state that there are positive and significant relationships between TRI and the four antecedents of behaviour intention in the UTAUT (performance expectancy, effort expectancy, social influence, and facilitating conditions) in the context of using wearable intelligent devices for sports and technology adoption in the classroom, respectively.

Other authors motivated by these ideas have proposed TRI as the basis for segmenting the samples studied. Devolder and colleagues [64], based on a sample of 204 Belgian users of an electronic patient record, detected technology readiness profiles by applying TRI. Then, they analysed the UTAUT model for each profile; their findings suggest the significant antecedents of the intention to use the system change from profile to profile. Recently, Hao and Chon [65] explored the relationships between customer experience, customer delight, customer equity, and brand trust in touchless hospitality services and the moderate effect of TRI on this phenomenon. Touchless hospitality services are technology-centric services that address the challenges of the COVID-19 pandemic. According to a multigroup analysis based on 1537 adult Chinese citizens, their results revealed effects of delight on equity and equity on trust are stronger for the consumer with a low TRI.

Inspired by these ideas of moderation in using information technologies based on generations and technological predisposition, we group the Chilean elderly users of social networking sites into three groups: independent, technologically apathetic, and technologically hungry. The independent group is composed of the generation of late baby boomers. These people have used, and some still use, information technologies in their working lives. Likewise, the massification of the Internet occurred when they were not even older people. The technologically apathetic group comprises the generations of silent and early baby boomers with technology readiness profiles of hesitators and avoiders. These persons did not use information technologies in their working lives, and the massification of the Internet occurred when they were already senior. They have a low degree of innovation and are highly resistant to technology adoption. Finally, the technologically hungry group includes generations of silent and early baby boomers with a technology readiness profile of explorers, pioneers, and sceptics. These individuals did not use information technology in their professional lives, and the massification of the Internet took place even though they were already older. Nevertheless, they are generally more likely to be attracted to technology, having minimal to medium resistance. According to this categorisation, we propose the following proposition:

**Proposition** **1** **(P1):** *There are statistically significant differences in the model of acceptance of social networking sites between the independent, technologically apathetic, and technologically hungry groups*.

## 3. Materials and Methods

### 3.1. Procedure and Participants

This study uses a quota sampling method to obtain data using strata: metropolitan area, generation, and gender. First, we determined the sample size with a stratified procedure by simple affixation but with a maximum allowed error of 2.5%. Using the formula of random sampling for finite samples and adopting a confidence level of 95%, the calculation of the global sample size was reduced to 1555 users. The global sample is distributed in the strata by simple affixation in the second step. This procedure ensures that the maximum allowed error does not exceed 5%, at a confidence level of 95%, for each stratum of the analysis domains (area, generation, and gender), according to the same simple random sampling formula for finite samples. Area: This study was conducted in Coquimbo and Biobío, two metropolitan areas of 506,000 and 1,009,000 inhabitants, respectively, in Chile. Both regions were considered since they have a higher proportion of the population over 60 in Chile. According to data from the National Institute of Statistics (INE), the proportion of older adults in Coquimbo is 16.7%, while in Biobío, it is 17.3%. As a sampling frame, the results of the most updated population census available in the country (INE-XIX National Population Census) for the regions of Coquimbo and Biobío were used. Generation: The population over 60 in Chile includes the silent generation (people born between 1927 and 1946), early baby boomers (individuals born between 1947 and 1955), and late baby boomers (people born between 1956 and 1961). Gender: To determine the proportion of Internet users by gender and generation, the results of the IX Survey of Internet Access and Use of the Undersecretary of Telecommunications of Chile were used, whose information constitutes the most up-to-date study available in the country. Table 1 shows the distribution of the stratified sample.

Paid interviewers conducted a face-to-face survey of older adults using social networks in places where older adults attend, such as social centres and health centres, in April 2022 in Coquimbo-La Serena conurbation and Concepción city. The interviewers were 23 health professionals and medical students trained explicitly before conducting the questionnaire.

All participants agreed to answer the questionnaire through informed consent voluntarily. The study was undertaken following the Declaration of Helsinki and endorsed by the Ethics Committee of the Universidad Católica del Norte (R05/2021).

### 3.2. Measures

Table 2 presents each latent variable included in the research model associated with the number of indicators that formed it and the source of the original English scale, and other studies that have validated this scale have been translated into Spanish in Chile, either for general or elderly users of information technologies. A five-point Likert scale was used for the questions in this study, with respondents choosing their responses on a scale ranging from total disagreement (1) to total agreement (5). The use of SNS was measured using formative indicators; all the other latent variables were measured using reflexive indicators. 

Additionally, the TRI was measured, and the technological predisposition profiles were calculated following Parasuraman and Colby [66]. The age of the respondent determined the generation.

**Table 2:** Summary of measurement scales.

Latent Variable	Number of Indicators	Original Scale in English	Scale in Spanish
Performance expectancy	4	[35]	[67]
Effort expectancy	3	[35]	[67]
Social influence	4	[35]	[68]
Facilitating conditions	4	[35]	[68]
Hedonic motivation	3	[35]	[67]
Habit	5	[35]	[68]
Intention to use	2	[35]	[68]
Use of SNS	4	[69]	[67]

Source: own elaboration.

### 3.3. Analysis of Results

Partial least squares structural equation modelling (PLS-SEM) was used to test the hypotheses. We selected PLS-SEM as the technique to examine the research model due to the existence of formative indicators to measure the latent variable use of SNS [70]. The guide developed by Henseler and colleagues [59] for PLS-SEM validation was used to reference the accepted levels of statistical values.

## 4. Results

### 4.1. Evaluation of the Measurement Model

The PLS-SEM is applied to both the measurement model and the structural model. As a previous stage of structural analysis, analysis of the reliability and validity of the measurement model is necessary. The results are provided below for the global sample and each group analysed.

Reliability was assessed by examining the individual loadings of the indicators with their respective latent variables. All loadings have values over the cutoff of 0.7; consequently, the results indicate appropriate reliability for all indicators. Cronbach’s alpha (CA) has been used as a reliability index for latent variables, and composite reliability (CR) was calculated. All the CR values are higher than the suggested minimum of 0.7. Therefore, all latent variables of the model have adequate reliability. The convergent validity of the latent variables was assessed through the inspection of the average variance extracted (AVE). AVE values exceed the upper limit value of 0.5 for all constructs, so it is possible to affirm that convergent validity is assured for all latent variables. Table 3 shows the indexes related to the latent variables.

The heterotrait–monotrait ratio of correlations (HTMT) was used to evaluate the discriminant validity of the constructs. Table 4 shows the results of this exam. All HTMT values are well below the recommended cutoff of 0.85, and thus all latent variables included in the model are conceptually distinct.

### 4.2. Evaluation of the Structural Model

To evaluate the structural model and estimate its parameters, the bootstrap procedure was applied with 5000 sub-samples. Table 5 summarises these results for the global sample and each group analysed.

As can be seen, for the global sample, all relationships included in the model are confirmed, except for the effort expectancy to the intention to use, since the corresponding *p*-values for effects are below the significance cut-off of 0.01. Therefore, all the hypotheses of the model except one of them are contrasted. Furthermore, for the global sample, the coefficients of determination show a good explanatory fit, with more than 50% of the variance in intention to use and 44% of the variance in the use of SNS being explained by the predictors of the model (R^2^ = 0.506 and R^2^ = 0.442, respectively). 

### 4.3. Multigroup Analysis 

Before carrying out the multigroup analysis, an analysis of the invariance of measurements was carried out using the MICOM procedure; its results indicate that it is possible to compare the path coefficients of the groups. The multipligroup analysis based on partial least squares (PLS-MGA) allows testing if pre-defined data groups have significant path coefficient differences. The results of PLS-MGA for the model with the three groups are presented in Table 6; the type test was two-tailed. As can be seen, there are two or three *p*-values for different effects below the significance cut-off of 0.05 among the groups. Particularly, these results supported proposition P1.

## 5. Discussion 

In this study, we explained the SNS acceptance in the elderly post-pandemic based on the UTAUT and TRI. Analysing a structural equation model and differences between elderly groups associated with different generations and technological predispositions show remarkable results.

Twelve years ago, Maier and colleagues [71] studied factors influencing the decision to adopt SNS among Germans aged 50 and older. Their results indicated that performance expectancy, social influence, effort expectancy, and fear of technology influence the intention to use in adopters; instead, performance expectancy and fear of technology influence the intention to use in SNS nonadopters. Contrary to these results but consistent with subsequent studies on the SNS [72,73,74,75], we found that effort expectancy has no direct effect on the intention to use the SNS. In general, we believe that the lack of relevance of this variable could be explained by the design sophisticated yet simple-to-use interface of the technology as well as its high penetration level, which places it as a daily tool for many people around the world, including elderly people. In particular, the study’s findings show that effort expectancy is not important in explaining the acceptance of consumer technologies in the elderly. Indeed, the relationship between effort expectancy and the intention to use SNSs is not significant for the overall sample. In the same way, among the elderly groups, this relationship is not significant. Again, these results are consistent with the previous literature regarding the overall sample [33], particularly in the post-pandemic era when we see the effect of effort expectancy on the intention to use technology by older people [38]. Currently, SNSs have been in our society for about fifteen years. Therefore, we could say that it is a mature technology with high penetration. Thus, the degree of ease associated with using the SNS is not an antecedent for the intention to use this technology.

Except for effort expectancy, the other antecedents of the intention to use SNS are significant in the acceptance model for the overall sample and all the elderly groups. All the antecedents of the use of SNS are significant for the entire sample and all the elderly groups, apart from the relationship between intention to use and use of SNS in the group labelled independent. This may mean that for the older adults surveyed, the interaction with this technology, as well as the ease of using it, is something that they have already learned and does not influence their intention to use it. Consequently, the other antecedents of the proposed model should be accomplished to encourage them to use SNS. In this sense, the availability of technological devices for accessing SNS, included in the facilitating conditions, continues to be an essential issue in the Chilean context. 

A recent study of the factors associated with adopting Internet search engines among older adults in Israel revealed that for the Silent Generation, only socioeconomic status and education define their adoption. On the other hand, all Baby Boomers’ sociodemographic characteristics were associated with using search engines [3]. In short, among younger older adults, a more significant number of variables explain their usage behaviour. In our result, which considers attitudinal variables, the number that defines the adoption of digital technology among older adults does not change between the youngest and the most senior, but the effect size of the explanations changes. In particular, concerning the results of each group of older adults, the findings are beneficial for carrying out interventions in each one of them in search of increasing the use of SNS and, possibly, of another type of consumer technology. Contrasting the P1 proposition indicates that heterogeneity in the use of social networks among the elderly in Chile is evident. Therefore, if the use of these technologies among the elderly is going to be promoted, different policies and actions are required for the three analysed groups because each group shows other behaviours in relation to the intention to use SNS. 

Although habit is the main explanation for SNS use in all groups, as in other studies [68,76], in the independent group, its effect is of such magnitude that the intention to use SNS is not a determinant of use. In this same group, the strength of facilitating conditions is essential to explain the intention and use of SNS. In fact, and associated with use, this effect is more than double that in the apathetic group. This means that it is essential to encourage the habit and mechanical behaviour of using social networks; once this is achieved, the use of these technologies is almost guaranteed. On the other hand, social influence affects each group differently; this effect approximately doubles in the independents compared to those technologically apathetic and those technologically hungry. Consequently, the extent to which a person perceives how vital others believe they should use this technology is much more critical for the late Baby Boomers than for the rest. Therefore, their social group could encourage this group of elders to utilise these technologies.

## 6. Conclusions

The main conclusion of this research is that we have explained the SNS acceptance in the elderly post-pandemic in the scope of Chile. We used two theoretical frameworks to achieve this purpose: the UTAUT2 and the TRI models. The main contributions are (1) showing how adults embrace consumer technologies after the pandemic; and (2) that it validates a classification of older adults who use helpful consumer technologies to understand the heterogeneity of this phenomenon.

### 6.1. Theoretical Implications

From a theoretical point of view, we can highlight some implications. First, the UTAUT2 model properly explains the acceptance of SNS by older Chileans. Most of the research on this topic has been carried out in other countries, mainly Western Europe, North America, and Southeast Asia. Second, to understand the heterogeneity of older people’s behaviour towards technology, psychographic characteristics have greater explanatory power than demographic characteristics. Third, we want to highlight that the only latent variable that does not explain technology use is effort expectancy, which may be due to several causes. For example, older people tend to have more free time, and their perception of effort expectancy may differ from that of younger people. Another cause may be that some social networks have already been established in society for over a decade. Therefore, people are already more familiar with these technologies, which reduces their perception of effort expectancy.

### 6.2. Practical Implications

From a practical point of view, we can highlight some implications. First, for developing strategies aimed at the independent group, it is necessary to consider measures that facilitate access to devices that provide access to SNS and offer a visualisation of these people in society. This would enable the development of a habit of using SNS. Second, according to our results, strategies targeting the technologically apathetic segment should be based on the ease of use of SNS and the individual satisfaction and benefit, hedonic, outcomes obtained by their participants. Finally, strategies designed for the technologically hungry older age group should focus on facilitating access to electronic devices to enjoy these technologies.

### 6.3. Limitations and Future Research

Although this study carried out a large sample size and with representative quotas of the population of older adults in Chile, it must be recognised as a limitation that the participants were not randomly selected. Therefore, in future studies, a random sampling method could be used to improve the generalisation of the results. On the other hand, although the results support generation as an essential variable to segment technological consumption by older adults, the generations used are typical of Chile. Consequently, extrapolating them to other countries without making the proper adjustments to the history of that nation is at least uncertain. We propose that future research in other countries should consider the inherent culture and sociodemographic features of the analysed country, for example, in Japan and western Europe, where these technologies are well-developed but the elderly have quite different cultures and sociodemographic characteristics.

## Figures and Tables

**Table 1:** The sample description.

Generation	Coquimbo		Biobío		Total by Gender		Total by Generation
	Women	Men	Women	Men	Women	Men	
Late Baby boomer	101	101	235	209	336	310	646
Early Baby boomer	104	67	182	122	286	189	475
Silent	79	64	173	118	252	182	434
Total	284	232	590	449	874	681	1555
Total area	516		1039				

Source: own elaboration.

**Table 3:** Cronbach’s alpha, composite reliability, and average variance extracted.

Latent Variable	Global Sample (*N* = 1555)			Independent (*N* = 646)			Technologically Apathetic (*N* = 460)			Technologically Hungry (*N* = 449)		
	CA	CR	AVE	CA	CR	AVE	CA	CR	AVE	CA	CR	AVE
Facilitating conditions	0.73	0.83	0.56	0.73	0.83	0.56	0.71	0.82	0.53	0.65	0.79	0.50
Effort expectancy	0.90	0.94	0.84	0.90	0.94	0.83	0.87	0.92	0.79	0.86	0.92	0.79
Performance expectancy	0.84	0.89	0.67	0.83	0.88	0.66	0.84	0.90	0.68	0.79	0.87	0.62
Habit	0.90	0.92	0.71	0.89	0.92	0.69	0.88	0.91	0.68	0.87	0.91	0.67
Intention to use	0.76	0.89	0.81	0.75	0.89	0.80	0.74	0.89	0.80	0.75	0.89	0.80
Hedonic motivation	0.93	0.95	0.88	0.91	0.95	0.85	0.94	0.96	0.89	0.92	0.95	0.87
Social influence	0.93	0.95	0.83	0.93	0.95	0.83	0.94	0.96	0.85	0.92	0.95	0.82

Source: own elaboration.

**Table 4:** Discriminant validity assessment.

	Latent Variable	FC	EE	PE	HA	IU	HM
Global sample	Effort expectancy	0.72					
	Performance expectancy	0.68	0.56				
	Habit	0.57	0.59	0.59			
	Intention to use	0.75	0.53	0.71	0.63		
	Hedonic motivation	0.67	0.51	0.63	0.62	0.69	
	Social influence	0.50	0.29	0.50	0.33	0.54	0.38
Independent	Effort expectancy	0.67					
	Performance expectancy	0.67	0.49				
	Habit	0.48	0.51	0.53			
	Intention to use	0.68	0.42	0.68	0.55		
	Hedonic motivation	0.60	0.43	0.65	0.51	0.61	
	Social influence	0.51	0.29	0.55	0.37	0.64	0.44
Technologically apathetic	Effort expectancy	0.65					
	Performance expectancy	0.67	0.55				
	Habit	0.54	0.52	0.58			
	Intention to use	0.72	0.50	0.71	0.66		
	Hedonic motivation	0.68	0.46	0.57	0.65	0.72	
	Social influence	0.52	0.35	0.46	0.26	0.47	0.31
Technologically hungry	Effort expectancy	0.66					
	Performance expectancy	0.55	0.45				
	Habit	0.47	0.50	0.55			
	Intention to use	0.76	0.49	0.63	0.59		
	Hedonic motivation	0.60	0.49	0.56	0.59	0.64	
	Social influence	0.53	0.27	0.52	0.40	0.53	0.39

Note. FC: facilitating conditions; EE: effort expectancy; PE: performance expectancy; HA: habit; IU: intention to use; HM: hedonic motivation. Source: own elaboration.

**Table 5:** Structural model results.

	Global Sample (*N* = 1555)		Independent (*N* = 646)		Technologically Apathetic (*N* = 460)		Technologically Hungry (*N* = 449)	
Relationship	Effect	*p*	Effect	*p*	Effect	*p*	Effect	*p*
Facilitating conditions -> intention to use	0.21	0.00	0.22	0.00	0.14	0.02	0.28	0.00
Facilitating conditions -> use of SNS	0.24	0.00	0.27	0.00	0.12	0.03	0.19	0.00
Effort expectancy -> intention to use	−0.01	0.58	−0.04	0.31	−0.01	0.86	0.02	0.72
Performance expectancy -> intention to use	0.18	0.00	0.17	0.00	0.21	0.00	0.14	0.01
Habit -> intention to use	0.17	0.00	0.16	0.00	0.17	0.00	0.15	0.00
Habit -> use of SNS	0.42	0.00	0.42	0.00	0.39	0.00	0.34	0.00
Intention to use -> use of SNS	0.13	0.00	0.04	0.38	0.20	0.00	0.22	0.00
Hedonic motivation -> intention to use	0.21	0.00	0.14	0.00	0.28	0.00	0.19	0.00
Social influence -> intention to use	0.17	0.00	0.26	0.00	0.13	0.00	0.14	0.00

Source: own elaboration.

**Table 6:** PLS-MGA results.

**Relationship**	**Apathetic–Hungry Effects**	***p***	**Apathetic–Independent Effects**	***p***	**Hungry–Independent Effects**	***p***
Facilitating conditions -> intention to use	**−0.15**	0.05	−0.08	0.29	0.07	0.40
Facilitating conditions -> use of SNS	−0.07	0.37	**−0.15**	0.02	−0.08	0.17
Effort expectancy -> intention to use	−0.02	0.71	0.03	0.64	0.05	0.35
Performance expectancy -> intention to use	0.07	0.37	0.04	0.50	−0.02	0.72
Habit -> intention to use	0.02	0.73	0.02	0.76	−0.01	0.93
Habit -> use of SNS	0.05	0.50	−0.03	0.60	−0.08	0.18
Intention to use -> use of SNS	−0.02	0.83	**0.16**	0.04	**0.18**	0.01
Hedonic motivation -> intention to use	0.08	0.32	0.14	0.07	0.06	0.43
Social influence -> intention to use	−0.01	0.97	**−0.13**	0.03	**−0.12**	0.04

Note. Significant differences in bold. Source: own elaboration.

## Data Availability

Not applicable.
